# Biological and chemical properties of five mineral oxides and of mineral trioxide aggregate repair high plasticity: an in vitro study

**DOI:** 10.1038/s41598-022-17854-0

**Published:** 2022-08-19

**Authors:** Amjad Abu Hasna, Lucas de Paula Ramos, Tiago Moreira Bastos Campos, Sergio Lucio Pereira de Castro Lopes, Maisour Ala Rachi, Luciane Dias de Oliveira, Cláudio Antonio Talge Carvalho

**Affiliations:** 1 Department of Restorative Dentistry, Endodontics Division, Institute of Science and Technology, São Paulo State University – UNESP, Av. Eng. Francisco José Longo Avenue 777, São José dos Campos, São Paulo CEP 12245-000 Brazil; 2 Department of Biosciences and Oral Diagnosis, Institute of Science and Technology, São Paulo State University – UNESP, São José dos Campos, São Paulo Brazil; 3 Physics Department, Aeronautics Technological Institute (ITA), São José dos Campos, São Paulo Brazil; 4 Department of Operative Dentistry, Syrian Private University (S.P.U), Damascus, Syria

**Keywords:** Microbiology, Materials science

## Abstract

Calcium silicate-based cements have diverse applications in endodontics. This study aimed to evaluate the antibiofilm action, biocompatibility, morphological structure, chemical composition and radiopacity of Five Mineral Oxides (5MO), Mineral Trioxide Aggregate Repair High Plasticity (MTA Repair HP), and Mineral Trioxide Aggregate (MTA) cements. MTT analysis was used to test the antibiofilm action of these cements against five anaerobic microorganisms, and test their biocompatibility with mouse macrophage (RAW 264.7) and osteoblasts (MG-63) cultures. Their morphological structure and chemical composition were evaluated by scanning electron microscopy (SEM) coupled to energy dispersion X-ray spectroscopy (EDX), and the phase analysis was performed by X-ray diffraction (XRD). Conventional radiography was used to assess the radiopacity of the cements. 5MO, MTA Repair HP and MTA were effective against *Porphyromonas gingivalis, Parvimonas micra, Fusobacterium nucleatum* and *Prevotella intermedia*, they were biocompatible with macrophages and osteoblasts after 5 min of contact, and they had adequate radiopacity to be used clinically. Bismuth oxide (Bi2O3) is used as a radiopacifier in MTA and 5MO, and calcium tungstate, in MTA Repair HP. Titanium dioxide (TiO_2_) (ANATASE) is responsible for the antimicrobial action and biocompatibility of 5MO.

## Introduction

Mineral Trioxide Aggregate (MTA) is a bioactive calcium silicate-based cement (CSC) derived from Portland cement^1^. It has been indicated for different clinical situations including apexogenesis, apexification, pulp revascularization and sealing of endodontic perforations^2–4^. Calcium hydroxide (Ca(OH)_2_) used to be the material of choice for these clinical situations^5^, until MTA was released.

New CSCs have been released in recent years, and have the same indications as MTA. Five Mineral Oxides CSC (5MO) is derived from Portland cement, developed to treat different types of dental accidents and endodontic complications, and is considered effective as a pulp capping material, a plug material in apicoectomies, and a perforation sealer^6–9^. Photodynamic therapy was indicated to be combined with the use of 5MO^7^ because of its antimicrobial action^10^. Recently, a new study showed that 5MO is effective over *P. gingivalis* and *P. endodontalis* beside to other anaerobic endodontic pathogens, and is not genotoxic over mouse macrophage (RAW 264.7) and osteoblast (Mg-63) cultures^11^.

More recently, Mineral Trioxide Aggregate Repair High Plasticity (MTA Repair HP) was released, representing a high plasticity CSC with improved physical properties^12^. It has proven biocompatibility and biomineralization^13^, and is cytocompatible with stem cells from human dental pulp^14^. It disinfects *P. micra*, however, its efficacy over other anaerobic endodontic pathogens is still questionable^11^. The first systematic review about MTA Repair HP recommended more long-term in vivo studies with a larger sample size and proper clinical settings to better understand its biological, chemical and physical properties^15^.

All these CSCs have demonstrated efficient clinical behavior proven in several studies^4,5,7,8,13^. However, the antimicrobial action, cytotoxicity, morphological structure, chemical composition, and radiopacity of Five Mineral Oxides (5MO) and MTA Repair HP have been little explored, if at all.

Therefore, the aim of this study was to investigate MTA, 5MO and MTA Repair HP in relation to: (I) their antimicrobial action against five strict anaerobic bacteria, including *Porphyromonas gingivalis, Porphyromonas endodontalis, Parvimonas micra, Fusobacterium nucleatum* and *Prevotella intermedia*; (II) their cytotoxicity to mouse macrophage (RAW 264.7) and osteoblasts (Mg-63) cultures; (III) their morphological analysis using scanning electron microscopy (SEM); (IV) their chemical composition established using energy dispersive x-ray spectroscopy (EDX) and X-ray diffraction (XRD); and (V) their radiopacity.

## Material and methods

### Antibiofilm activity

Five different inocula of *P. gingivalis* (ATCC 33277), *P. endodontalis* (ATCC 35406), *P. micra* (ATCC 23195), *F. nucleatum* (ATCC 25586) and *P. intermedia* (ATCC 33563) were prepared, standardized in saline solution (NaCl 0.9%) (Eurofarma, São Paulo, SP, Brazil) and at 1 × 10^8^ CFU/mL in a spectrophotometer (Visible Spectrophotometer V-5000, Shanghai Metash Instruments, China). Then, 100 µl of corresponding inoculum was added to each well of 96-well microplates. The plates were incubated (37 °C) under agitation (75 rpm) for 90 min. Afterwards, the supernatant was discarded, 100 µL of Brucella broth (Himedia, Mumbai, India) was added, and the plates were incubated (37 °C) for 7 days in anaerobic conditions; the culture medium was replaced every 48 h. After the biofilms were formed, the materials were placed in contact with the biofilms to be evaluated for 24 h, and the plates were incubated (37 °C) in anaerobic conditions. Subsequently, the biofilm measurement test (MTT) was performed. Two independent experiments were carried out with 5 repetitions each, totaling n = 10 for each experimental group.

The experimental groups were: (I) saline solution (negative control group) (Eurofarma, São Paulo, SP, Brazil); (II) 2.5% sodium hypochlorite (NaOCl) (Biodynamics, Ibiporã, PR, Brazil) (positive control group); (III) 5MO (Golden Yatti LLC, Muscat, Oman); (IV) MTA Repair HP (Angelus, Londrina, PR, Brazil); and (V) MTA (Angelus, Londrina, PR, Brazil). All the CSCs were spatulated according to the proportions specified in the manufacturer's instructions (3 × Powder: 1 × liquid) and applied immediately in a fresh state. After 24-h contact with the biofilms, the CSCs were analyzed by adding 100 µL of MTT solution (3-[4,5-dimethylthiazol-2-yl]-2,5-diphenyltetrazolium bromide) (Sigma-Aldrich, St. Louis, MO., USA). The plates were incubated, while protected from light for 1 h in an anaerobic chamber at 37 °C. Then, the MTT solution was removed, 100 µL of dimethyl sulfoxide (DMSO) (Sigma-Aldrich, St. Louis, MO., USA) was added, the plates were incubated again at 37 °C for 10 min, and placed on a constant shaker for 10 min. Lastly, the plates were read in a microplate reader at 570 nm (BIO-TEK Instruments, Highland Park, Winooski, VT, USA). The optical densities (OD) obtained were converted into a percentage of microorganism cell viability using the following formula:

$$\% {\text{Viability}} = \left( {{\text{OD of Treated Group}} \times 100} \right)/{\text{Mean OD of Control Group}}).$$

### Cytotoxicity analysis

Cultures of mouse macrophages (RAW 264.7) (Rio de Janeiro Cell Bank - APABCAM, RJ, Brazil) and osteoblasts (MG-63) (Rio de Janeiro Cell Bank - APABCAM, RJ, Brazil) were used. The cells were kept in cell culture flasks (TPP, Zollstrasse, Switzerland), and grown in Dulbecco’s Modified Eagle Medium (DMEM) (LGC Biotechnology, Cotia, SP, Brazil), supplemented with 10% fetal bovine serum (FBS) (Invitrogen, Grand Island, NY, USA), incubated at 37 °C, with atmospheric humidity, and 5% CO_2_. The culture medium was changed every 48 h, and the cells were transferred to another cell flask when a state of cell subconfluence of the cells was observed. The cells were transferred to a Falcon tube where they were centrifuged for 5 min at 2000 rpm. The number of viable cells was quantified by the Trypan blue exclusion test (0.4%, Sigma-Aldrich, St. Louis, MO, USA). The cells were cultivated in 96-well microplates. A 200-µl aliquot of DMEM was added and supplemented with 10% FBS containing 2 × 10^4^ viable cells, after which the plates were incubated (37 °C, 5% CO_2_) for 24 h to promote cell adhesion. Then, the supernatant was discarded, and the cells were washed with PBS. Control wells containing only cells with culture medium were used. The incubation period was 5 min and 24 h, and there were 12 wells per group (n = 12).

The CSCs were manipulated in a 24-well plate following the manufacturer's instructions. Additional group of Ca(OH)_2_ (Biodynamic Chemicals and Pharmaceuticals, Ibiporã, PR, Brazil) was tested. It was manipulated using a powder to liquid proportion of 1:1. The materials were mixed, and each well containing the respective cement was filled with 2 mL of culture medium (DMEM) supplemented with 10% FBS, after which the plates were incubated at 37 °C for 24 h with 5% CO_2_.

Then, 100 µL per well of the conditioned DMEM from each experimental group was applied to the cells. The NaOCl group was used as the control. The viability of the culture was determined by analyzing the mitochondrial activity of viable cells using MTT reduction in formazan, where 100 µL of MTT solution was added per well, and the plates were incubated (37 °C, 5% CO_2_) for 1 h, protected from light. This solution was then discarded, and 100 µL of DMSO was added per well to expose the formazan crystals produced after the viable cells absorbed the MTT salt. After the wells were incubated for 10 min and shaken for the same amount of time, their absorbance was read in a microplate reader with a wavelength of 570 nm. The OD obtained was converted into a percentage of cell viability using the following formula:

$$\% \,{\text{Viability}} = \left( {{\text{OD of Treated Group}} \times 100} \right)/{\text{Mean OD of Control Group}}.$$

### Morphological analysis and chemical composition of the groups analyzed by using energy dispersion X-ray spectroscopy (EDX) and X-ray diffraction (XRD)

These analyses were performed on CSC discs manipulated according to the manufacturer’s instructions, compacted between two glass plates in silicone molds (5 mm × 2 mm) until the cements were fully set. The discs were placed in Petri dishes and stored at 100% humidity for 3 days at 37 °C, after which they were either (a) maintained at 100% humidity at 37 °C (control), or (b) immersed in distilled water for 4 h followed by air drying for 12 h.

All the stubs were coated with carbon to promote electrical conductivity. Samples were visualized by SEM (MIRA3-TESCA, Brno-Kohoutovice, Czech Republic). Images of the different components of the microstructure of the material were captured at different magnifications up to 100 k × in electron backscatter mode.

The chemical composition of the discs was determined by EDX using SEM. Phase analysis was performed on samples from each experimental group, using XRD by Cu Kα radiation and a double-crystal monochromator, in an automated powder diffractometer. The samples were presented in powder form, in a single crystal sample holder, which was used to avoid unwanted diffraction peaks. Phase identification was performed by using search matching software available from the International Center for Diffraction Data (ICDD) database.

### Radiopacity analysis

The radiopacity of the CSCs was evaluated using an aluminum step-wedge scale with increasing thickness (1 to 8 mm). The CSC discs were compacted into 5-mm diameter, 2-mm high silicone rings. The CSC discs and the aluminum scale were fixed with adhesive tape on a charge-coupled device (CCD) sensor of a teleradiographic device, to ensure standardization of the distance between the image receiver and the X-ray source, and to obtain a digital image. The radiographic device used was an ORTHOPHOS XG 5 (Dentsply Sirona, York, PA, USA) operating with acquisition parameters of 60 kVp, 10 mA, and exposure time of 9.1 s. The image was produced from a single acquisition, and submitted to pixel intensity analysis in an image analysis program (ImageJ 1.53e, Wayne Rasband and Contributors, National Institute of Health, USA).

Regions of interest corresponding to the images of the cements and of the steps of the aluminum stepwedge scale were determined with the selection tool, and were analyzed individually and progressively to obtain the histogram of pixel value distribution (mean, standard deviation, maximum and minimum values). A comparative analysis was then carried out between the mean values for the cements and those corresponding to the steps of the aluminum stepwedge scale, to determine the closest equivalence between the density of the studied materials and the thickness of the aluminum (in mm).

### Statistical analysis

All the data were submitted to a normality test, and then analyzed with the Kruskal Wallis test, complemented by the Dunn test, considering a significance level set at α ≤ 0.05, using GraphPad Prism 6 (La Jolla, CA, USA).

## Results

### Antibiofilm activity

**Figure 1:**
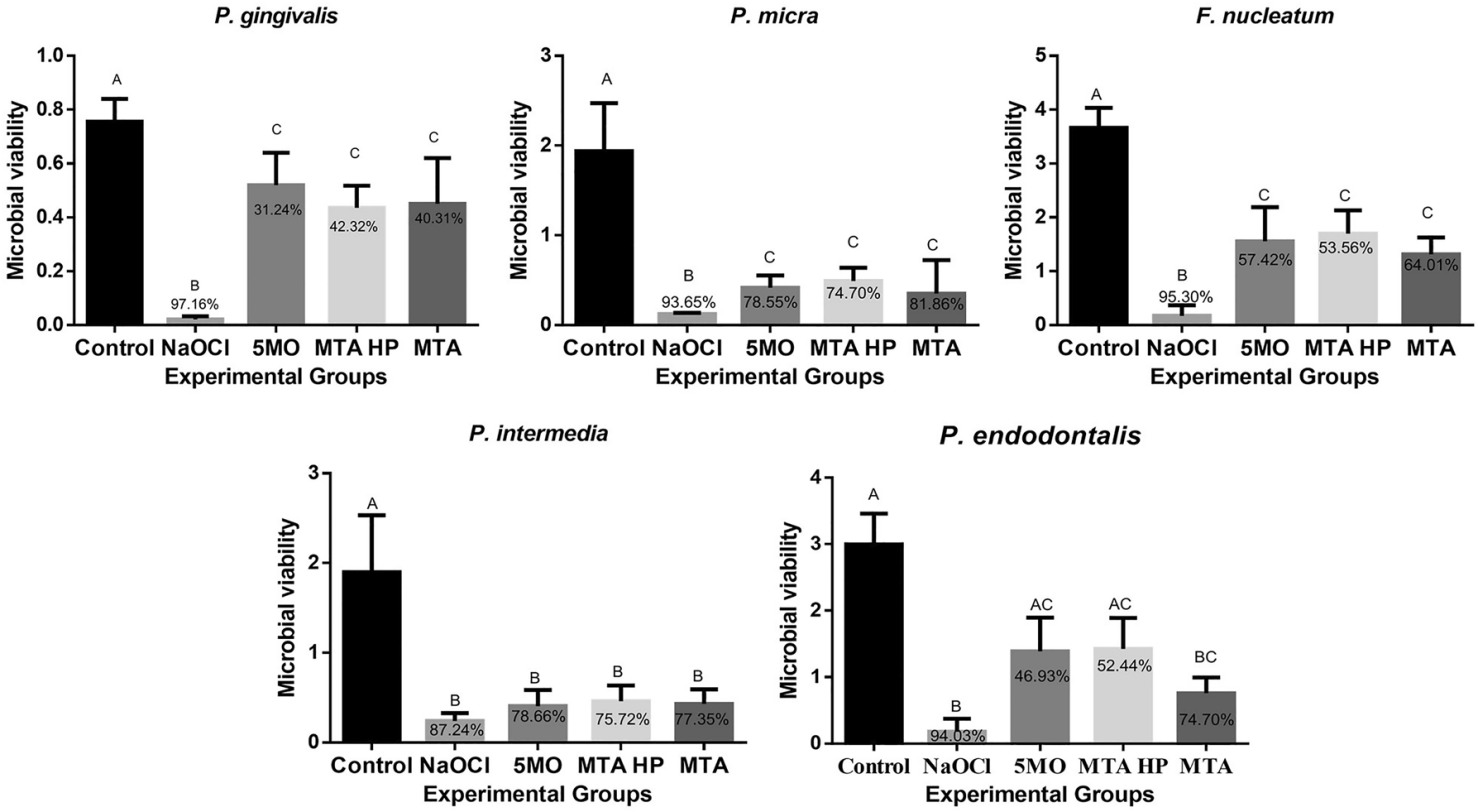
Microbial viability calculated by reflectance and viability reduction (in%) of *P. gingivalis*, *P. endodontalis*, *P. micra*, *F. nucleatum*, and *P. intermedia* biofilms using the MTT test after treatment with the experimental groups. Uppercase letters indicate a statistical difference.

All the CSCs were effective against all the microbial biofilms evaluated, resulting in a statistically significant reduction compared with the control group (*P* < 0.0001), except against *P. endodontalis* where MTA was similar to the NaOCl group and different of the control group (*P* < 0.0001), but 5MO and MTA Repair HP were similar to the control group (*P* > 0.05) (Fig. 1).

The viability reduction of all the microbial biofilms evaluated, except of *P. intermedia*, exceeded 90% with NaOCl group. However, this reduction varied between 31.24–78.66%, 42.32–75.72%, and 40.3–81.86% with 5MO, MTA Repair HP and MTA groups, respectively (Fig. 1).

### Cytotoxicity analysis

**Figure 2:**
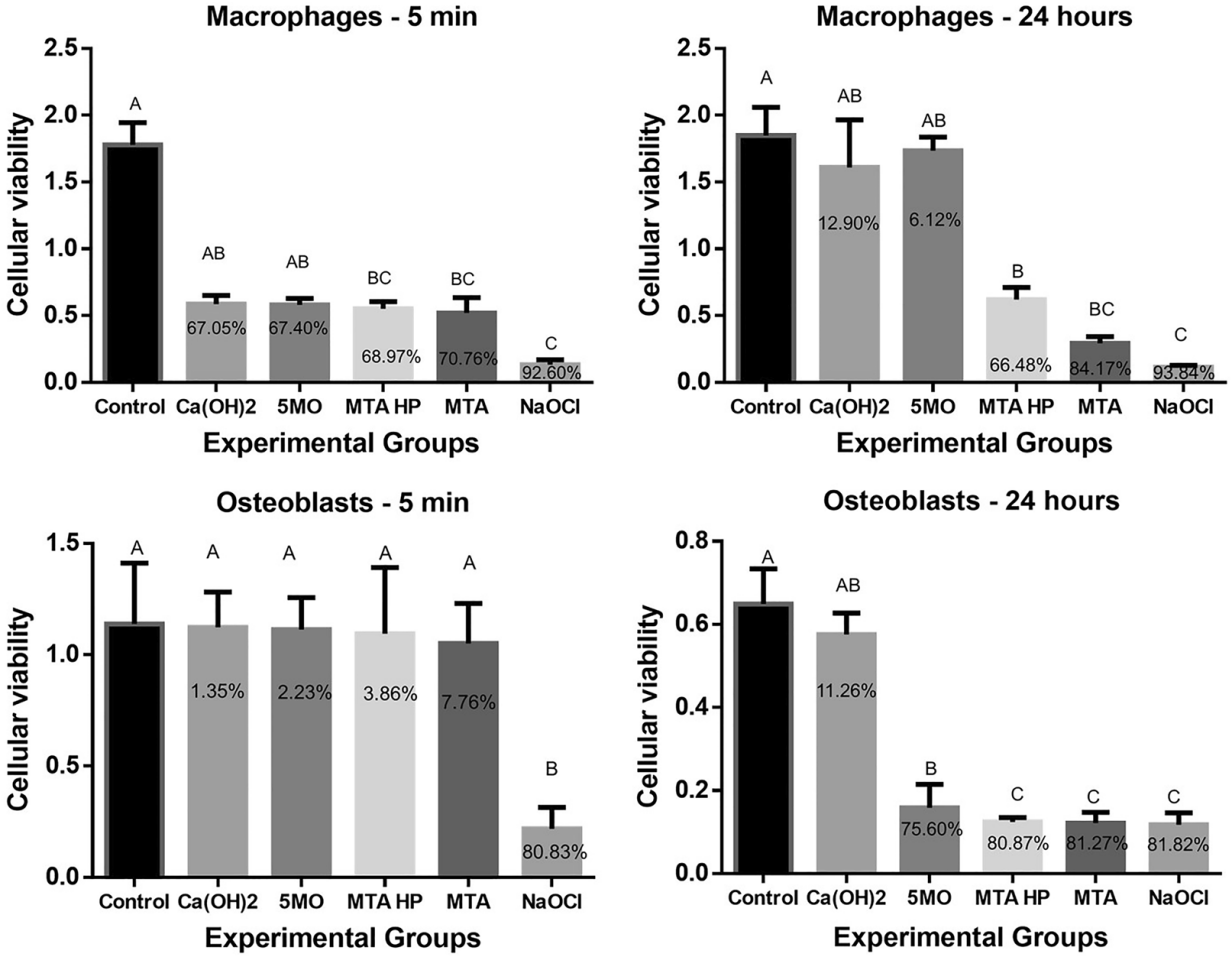
Cellular viability calculated by reflectance and viability reduction (in%) in macrophage (RAW 264.7) and osteoblast (Mg-63) cultures using the MTT test after treatment with the experimental groups. Uppercase letters indicate a statistical difference.

The Ca(OH)_2_ and 5MO groups showed a statistical significant difference (*P* < 0.0001) from the NaOCl group with macrophages, after 5 min and 24 h. There was no significant difference among the Ca(OH)_2,_ 5MO, MTA Repair HP and MTA groups (*P* > 0.05). Ca(OH)_2_ and 5MO had increased biocompatibility after 24 h. All experimental groups were biocompatible with osteoblasts after 5 min, except the NaOCl group. However, after 24 h, only Ca(OH)_2_ and 5MO groups were statistically different (*P* < 0.0001) from the NaOCl group (Fig. 2).

The viability reduction of macrophages and osteoblasts ranged between 80.83 and 93.84% with NaOCl group. However, the other groups were more biocompatible, the reduction varied between 1.35–67.05%, 2.23–75.60%, 3.86–80.87%, and 7.76–84.17% with Ca(OH)_2_, 5MO, MTA Repair HP and MTA groups, respectively (Fig. 2).

### Morphological analysis and chemical composition of the groups analyzed by using EDX and XRD

**Figure 3:**
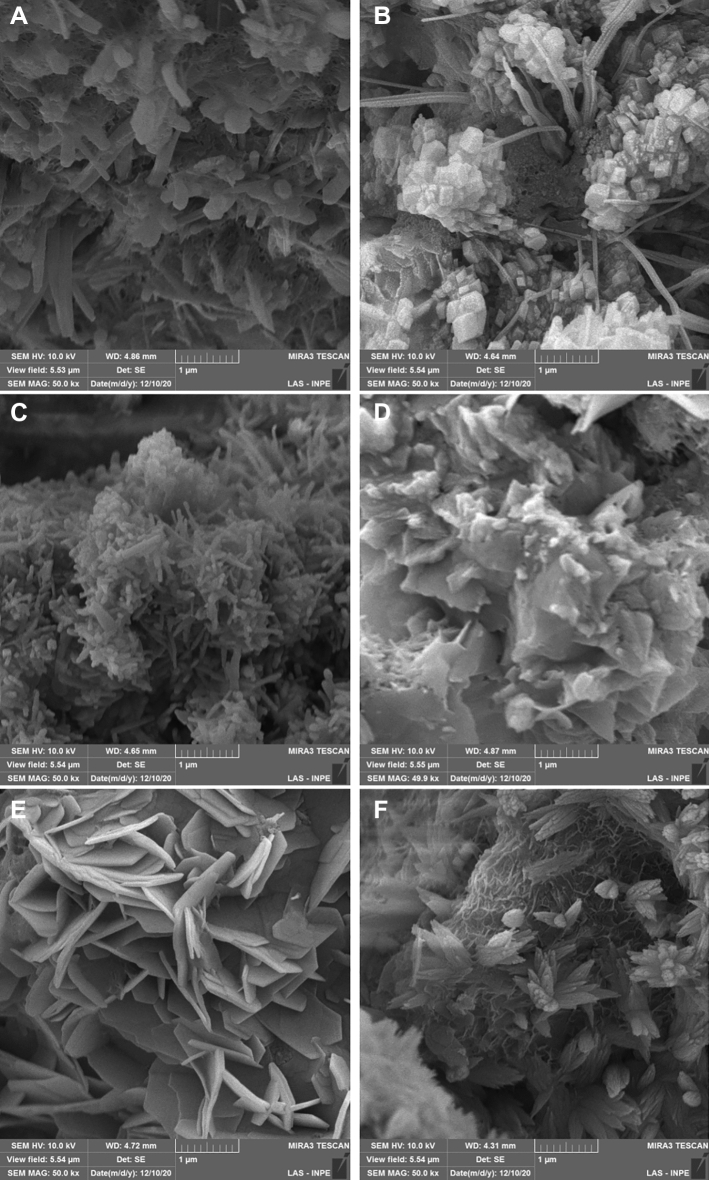
Scanning electron microscopy images (50 K ×) of Five Mineral Oxides (5MO) discs (**A**) maintained at 100% humidity and 37 °C (control); (**B**) immersed in distilled water for 4 h followed by air drying for 12 h; Mineral Trioxide Aggregate Repair High Plasticity (MTA Repair HP) (**C**) maintained at 100% humidity and 37 °C (control); (**D**) immersed in distilled water for 4 h followed by air drying for 12 h; and Mineral Trioxide Aggregate (MTA) (**E**) maintained at 100% humidity and 37 °C (control); (**F**) immersed in distilled water for 4 h followed by air drying for 12 h.

SEM images showed small irregular particles interspersed with some elongated needle-like particles in the 5MO group, small irregular particles with some larger particles and elongated particles in the MTA Repair HP group, and crystalline particles in the MTA group (50 k ×) (Figs. 3). The EDX showed the principal components of each CSC (Table 1).

**Table 1:** The chemical components of Five Mineral Oxides (5MO); Mineral Trioxide Aggregate Repair High Plasticity (MTA Repair HP); and Mineral Trioxide Aggregate (MTA) after EDX analysis.

Material	Chemical components
5MO	Ca, O, Bi, C, Ti, Si, Al, S, Mg, K and Fe
MTA Repair HP	Ca, O, W and Sb
MTA	Ca, O, Bi, Si, C and Al

The presence of titanium, sulfur and potassium, and the absence of tungsten in 5MO makes it different from MTA and MTA Repair HP, and the presence of tungsten and antimony in MTA Repair HP makes it different from 5MO and MTA (Table 1).

**Figure 4:**
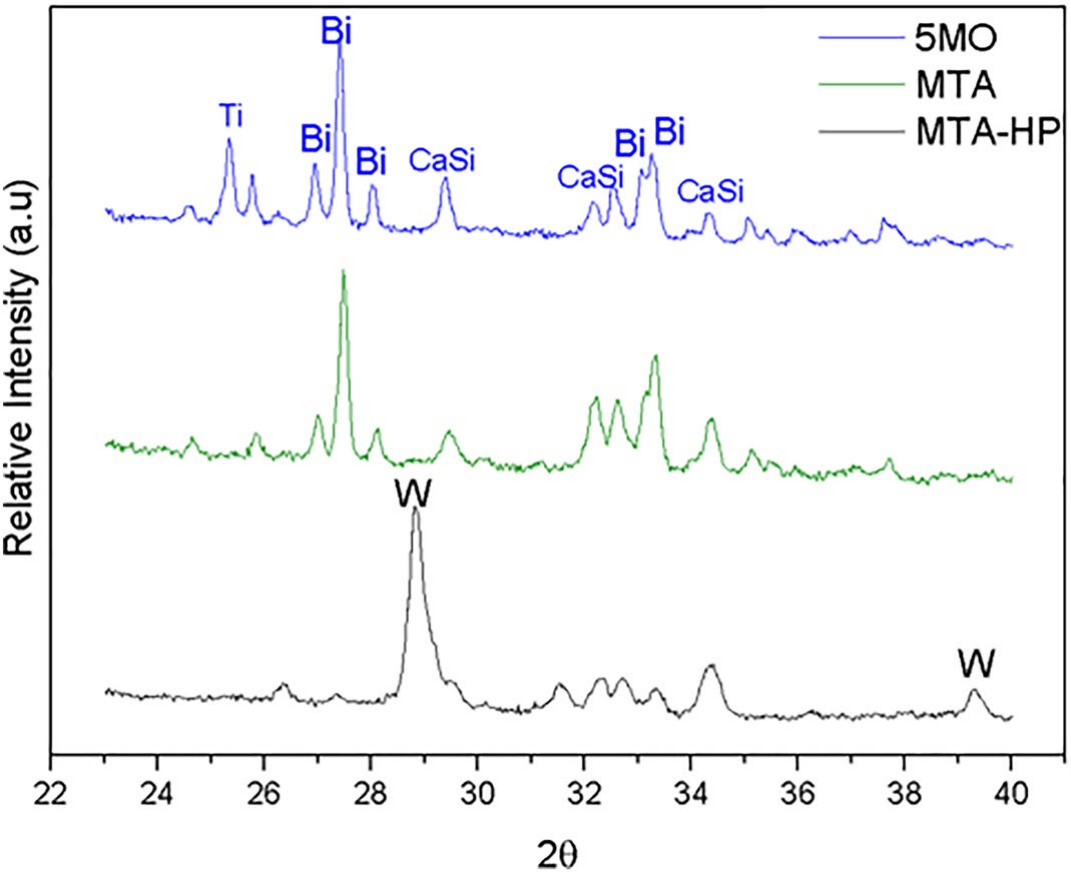
Phase analysis of the CSC cements by XRD. (Bi = Bi2O3, CaSi = Ca3O5Si [alite], Ti = TiO_2_ [anatase], W = CaWO4).

Phase analysis showed that the main difference between 5MO and MTA is the presence of titanium dioxide (TiO2) (Anatase). Both 5MO and MTA contain bismuth oxide (Bi2O3) and tricalcium silicate (Ca3O5Si) (Alite). On the other hand, MTA Repair HP contains calcium tungstate (CaWO4) (Fig. 4).

### Radiopacity analysis

The histograms of the CSCs showed that 5MO radiopacity corresponded approximately to the 7th–8th steps of the aluminum scale (7–8 mm of aluminum; mean between the two steps); MTA Repair HP has a radiopacity corresponding to the 8th step (8 mm) of the aluminum scale, thus exceeding the average for this step; MTA has a radiopacity corresponding to the 6th step (6 mm) of the aluminum scale.

## Discussion

MTA is a CSC with proven antimicrobial action, based on some studies reported in the literature. According to Torabinejad et al.^16^, MTA has an antibacterial effect on some facultative bacteria; however, it has no effect on strict anaerobic bacteria, compared with the effect of amalgam, zinc oxide-eugenol, and Super EBA on these bacteria. MTA antimicrobial action varies according to its concentration^17^; this somewhat explains the controversial results reported in the literature^18^. In the present study, MTA was observed as having effective antimicrobial action against all the microorganisms tested, given that it significantly reduced the biofilm viability. El Reash et al.^19^ cautioned that the antimicrobial action of CSCs against strictly anaerobic bacterial species is still questionable, thereby putting into evidence the pioneer and innovative nature of the findings of the present study and their relative contribution.

MTA Repair HP has antimicrobial action against *E. faecalis*^20^; however, a more recent study showed that although MTA Repair HP did not inhibit the planktonic growth of *S. mutans*, it did reduce *S. mutans* biofilm formation^21^. In the present study, MTA Repair HP was effective against all microorganisms evaluated except *P. endodontalis*. The antimicrobial action of 5MO has been little explored, a recent study showed its efficacy over *P. micra*, *P. gingivalis* and *P. endodontalis*^11^; for now, 5MO has been indicated in photodynamic therapy to ensure adequate disinfection^7^. This indication also applies to other CSCs when their antimicrobial action is questionable^22^. In the present study, 5MO showed effective antimicrobial action against all microorganisms evaluated in the MTT test, except *P. endodontalis.* Furthermore, NaOCl was used as a control group because of its antimicrobial action against several microorganisms reported in the literature^23^.

MTA is less toxic to mouse L929 cells than amalgam, Super EBA, and IRM^24^. It has been indicated for clinical use ever since it showed biocompatibility with MG63 osteoblasts^25^, and this biocompatibility has been reported to increase after 5 days^26^. In the present study, all the CSCs were found to be biocompatible with MG63 osteoblasts after 5 min, but only 5MO remained biocompatible after 24 h. MTA is also biocompatible with macrophages^27^, and its biocompatibility increases after 1 and 2 days^28^. In the present study, MTA was biocompatible with macrophages, and there was no significant difference among the Ca(OH)_2_, 5MO, MTA Repair HP and MTA groups after 5 min and 24 h.

Likewise, MTA Repair HP has biocompatibility similar to that of MTA with L929 fibroblasts, human dental pulp stem cells, and osteoblasts after 24, 48, and 72 h, and this biocompatibility increases over time^13^. Differently, in another study, the cellular viability of osteoblast decreased significantly after 72 h of contact with diluted MTA Repair HP^29^. The outcomes of the last study agree in somehow with the present study, where it was found that MTA Repair HP became biocompatible only after 5 min, and after 24 h this biocompatibility decreased These controversial results can be explained by the pH of the cement at the time of application and by the cement concentration^29,30^.

The biocompatibility of 5MO has not yet been studied; therefore, the results presented herein make this a pioneer study with a relevant contribution to endodontics, given that the biocompatibility of 5MO was similar to that of Ca(OH)_2_ and the control groups after 5 min of application on mouse macrophages, and similar to that of Ca(OH)_2_, MTA Repair HP and MTA groups after 24 h. Furthermore, the 5MO cement group was not significantly different from any other group evaluated, except the NaOCl group after 5 min of contact with osteoblasts, and was similar to the Ca(OH)_2_ group after contact for 24 h.

MTA is derived from Portland cement and contains bismuth oxide as a radiopacifier^31^. According to the manufacturer (Angelus), the main difference between MTA and MTA Repair HP is that the bismuth oxide radiopacifier composing the former was replaced by calcium tungstate in the latter, to ensure against dental discoloration. This result was corroborated in the XRD analysis of the present study. The study also showed that MTA has a chemical phase of Alite and bismuth oxide. This result is in agreement with that reported in the literature^32,33^. Furthermore, the composition and morphology of MTA observed in our study appeared in elongated and needle-like particles, and was influenced by environmental pH, the presence of ions, and contamination with blood, also corroborating the findings of the literature^34,35^. Nevertheless, the crystallinity presented by CSC after being manipulated was affected by its hydration^36^.

In the literature, EDX analysis revealed that MTA Repair HP have carbon, calcium and oxygen just like MTA and Biodentine^14,37^. However, in the present study, only calcium and oxygen were found in addition to tungsten and antimony. These divergent findings may be explained by the date of the studies, as the chemical formula of the cements may suffer alterations, still, further studies are needed to clarify the divergence. In the present study, calcium tungstate (CaWO4), the radiopacifier, was found in XRD analysis in MTA Repair HP, similarly, it was also found in another study associated with calcium silicates Ca3SiO5 and Ca2SiO4^38^.

To the best of our knowledge, there is no study on the chemical composition of 5MO. The present study found titanium, sulfur and potassium, but no tungsten in the 5MO cement, which has a different chemical composition from MTA and MTA Repair HP; it also found antimony in MTA Repair HP, thus making it different from 5MO and MTA. The XRD analysis revealed the presence of Anatase in 5MO, a compound that has antimicrobial activity^39^, and is biocompatible^40^, thus explaining the results of 5MO in relation to the other cements, as reported in the present study and corroborated by the literature.

In general, all CSCs have a radiopacity greater than that of dentin^41^, in compliance with the protocol for radiopacity of dental materials, published by the International Standards Organization (ISO)^42^. In the present study, all the CSCs showed adequate radiopacity: 5MO radiopacity corresponded approximately to the 7th–8th steps of the aluminum scale (7–8 mm of aluminum; mean between the two steps); MTA Repair HP had a radiopacity corresponding to the 8th step (8 mm) of the aluminum scale, and exceeded its average; MTA has a radiopacity corresponding to the 6th step (6 mm) of the aluminum scale. In the literature, three studies reported no significant difference between MTA and MTA Repair HP radiopacity^29,43,44^. In the present study, no statistical analysis were made as the samples were illustrative, still, all the tested cements presented radiopacity within the specifications of the American National Standard Institute/American Dental Association.

## Conclusions

5MO, MTA Repair HP and MTA cements: (I) have effective antimicrobial action against *P. gingivalis, P. micra, F. nucleatum* and *P. intermedia* biofilms, and (II) are biocompatible with macrophages and osteoblasts after 5 min [5MO showed increased biocompatibility with macrophages after 24 h, compared with the other cements]; and (III) have an adequate degree of radiopacity. Furthermore, bismuth oxide is the radiopacifier contained in MTA and 5MO, calcium tungstate is the radiopacifier contained in MTA Repair HP, and anatase is responsible for the antimicrobial action and biocompatibility of 5MO.

## Data Availability

The data used to support the findings of this study are available upon request with the corresponding author d.d.s.amjad@gmail.com.
